# Neuromuscular exercise reduces low back pain intensity and improves physical functioning in nursing duties among female healthcare workers; secondary analysis of a randomised controlled trial

**DOI:** 10.1186/s12891-019-2678-x

**Published:** 2019-07-13

**Authors:** Annika Taulaniemi, Markku Kankaanpää, Kari Tokola, Jari Parkkari, Jaana H. Suni

**Affiliations:** 1 UKK Institute for Health Promotion Research, Kaupinpuistonkatu 1, 33500 Tampere, Finland; 2 Department of Physical and Rehabilitation Medicine, Tampere University Hospital, Tampere, Finland

**Keywords:** Spinal pain, Recurrent low back pain, Sub-acute low back pain, Pilates, Nursing personnel, Exercise intervention, Movement control impairment

## Abstract

**Background:**

Low back pain (LBP) is common among healthcare workers, whose work is physically strenuous and thus demands certain levels of physical fitness and spinal control. Exercise is the most frequently recommended treatment for LBP. However, exercise interventions targeted at sub-acute or recurrent patients are scarce compared to those targeted at chronic LBP patients. Our objective was to examine the effects of 6 months of neuromuscular exercise on pain, lumbar movement control, fitness, and work-related factors at 6- and 12-months’ follow-up among female healthcare personnel with sub-acute or recurrent low back pain (LBP) and physically demanding work.

**Methods:**

A total of 219 healthcare workers aged 30–55 years with non-specific LBP were originally allocated to four groups (exercise, counselling, combined exercise and counselling, control). The present study is a secondary analysis comparing exercisers (*n* = 110) vs non-exercisers (*n* = 109). Exercise was performed twice a week (60 min) in three progressive stages focusing on controlling the neutral spine posture. The primary outcome was intensity of LBP. Secondary outcomes included pain interfering with work, lumbar movement control, fitness components, and work-related measurements. Between-group differences were analysed with a generalised linear mixed model according to the intention-to-treat principle. Per-protocol analysis compared the more exercised to the less exercised and non-exercisers.

**Results:**

The mean exercise attendance was 26.3 (SD 12.2) of targeted 48 sessions over 24 weeks, 53% exercising 1–2 times a week, with 80% (*n* = 176) and 72% (*n* = 157) participating in 6- and in 12-month follow-up measurements, respectively. The exercise intervention reduced pain (*p* = 0.047), and pain interfering with work (*p* = 0.046); improved lumbar movement control (*p* = 0.042), abdominal strength (*p* = 0.033) and physical functioning in heavy nursing duties (*p* = 0.007); but had no effect on other fitness and work-related measurements when compared to not exercising. High exercise compliance resulted in less pain and better lumbar movement control and walking test results.

**Conclusion:**

Neuromuscular exercise was effective in reducing pain and improving lumbar movement control, abdominal strength, and physical functioning in nursing duties compared to not exercising.

**Electronic supplementary material:**

The online version of this article (10.1186/s12891-019-2678-x) contains supplementary material, which is available to authorized users.

## Background

In the majority (85–90%) of people with low back pain (LBP), the pain is classified as non-specific low back pain (NSLBP) [1]. The traditional assumption is that after an episode of acute pain, most recover spontaneously within 6 weeks [2]. This assumption has been criticised [3], as LBP is often a long-term or recurrent condition wherein individuals experience repeated episodic back pain that comes and goes over an extended span of time [4, 5]. LBP becomes chronic in 10% of sufferers [6].

LBP is the leading and most costly musculoskeletal disorder among healthcare workers [7, 8]. The one-year prevalence of LBP among nursing personnel varies from 45 to 77% [7, 9–11]. Healthcare workers are exposed to physically heavy work duties, like lifting and transferring patients and prolonged standing or working in a stooped position, which are biomechanical risk factors for LBP and chronic pain [12–15].

Exercise is the most frequently recommended treatment for NSLBP [6, 16, 17]. However, exercise interventions targeted at sub-acute patients are scarce compared to those targeted at chronic LBP patients. There is moderate-quality evidence that post-treatment exercise can reduce the recurrence of back pain [18], and leisure-time physical activity can be beneficial in preventing low back pain [19]. However, the results of exercise treatment studies are conflicting, and it is difficult to specify the content of an effective programme [6].

LBP tends to affect and change motor behaviour [20]. Impairments in postural and movement control of the lumbar spine have been posited to be risk factors for prolonged LBP [21, 22]. A significant difference in the ability to actively control the movement of the low back has been found between patients with LBP and healthy subjects [23]. Female nurses with a recent back injury show more impairments in lumbar control compared to healthy nurses [14]. Both hypo- and hyper-lordosis correlate with degenerative joint disease, particularly in women [24]. Lumbar movement control, especially control of the lumbar neutral spine posture, has been suggested to play a key role in maintaining a healthy spine [25]. However, it is still unclear whether poor lumbopelvic control is a cause for LBP or a consequence of it. Evidence on the effects of movement control exercise interventions on pain intensity is only small to moderate [26, 27].

There is increasing evidence that low performance levels for different components of physical fitness are risk factors for LBP [28, 29], and a self-reported low rating of physical capacity is a predictor for future LBP in female healthcare workers [30]. Evidence about those associations is still partly conflicting with respect to revealing whether physical inactivity and deconditioning cause LBP or, alternatively, LBP leads to decreased physical activity and deconditioning [31]. Among the participants of the present study, high cardiorespiratory and muscular fitness were strongly associated with lower baseline medical costs and sickness-related absences [32].

Spinal stability and control of the spine [33] are considered to be important for back health [34]. Different approaches to exercising have been emphasised to achieve spinal stability; however, no single approach has proved to be superior [6, 35, 36].

Pilates is aimed at spinal alignment and a neutral spine posture [37]. It has been defined as “a mind-body exercise that targets core stability, strength, flexibility, posture, breathing, and muscle control” [38]. The exercises are often considered to be similar to spinal stabilisation / motor control exercises; however, they do not involve conscious activation of specific deep core muscles in the manner often used in spinal stabilisation exercises [39]. However, there is inconclusive evidence that Pilates is superior to other forms of exercise in reducing pain and disability in people with LBP [39]. Studies report a reduction in chronic LBP [39, 40], but to our knowledge, no studies investigating the effects of Pilates for people with non-chronic (sub-acute or recurrent) LBP have been reported. In a blinded four-arm randomised controlled trial (RCT; combined neuromuscular exercise and back care counselling, exercise only, counselling only, and controls), Suni and colleagues [41] found that combined neuromuscular exercise (NME) and back care counselling was effective in reducing LBP and related sickness absence and work-related fear of pain in female healthcare personnel with recurrent LBP. The present study aims to investigate the effectiveness of this 6 months Pilates-type NME with emphasis on control of the lumbar neutral zone of the above RCT in two-arm design i.e. NME and non-NME. More specifically, the study examines the effectiveness of NME on pain intensity and pain interfering with work, lumbar movement control impairments (MCI), fitness components, and work-related factors immediately after the intervention and at a 12-month follow-up in female healthcare personnel with sub-acute or recurrent LBP. We hypothesised that NME reduces LBP intensity and pain interfering with work, and improves lumbar movement control, fitness levels, and work-related factors more than non-exercise [42].

## Methods

### Study design and participants

This study is a secondary analysis of the four-arm randomised controlled trial “Neuromuscular exercise and back care counselling for female nursing personnel with recurrent non-specific low back pain: study protocol of a randomised controlled trial (NURSE RCT, clinical trial registration NCT01465698)”, in which healthcare workers with sub-acute or recurrent LBP were randomised to participate in supervised neuromuscular exercise or non-exercise and to receive back care counselling or non-counselling for 6 months [42].

The NURSE RCT was conducted in three consecutive sub-studies to achieve an adequate sample size [41]. The participants were female healthcare workers in physically demanding nursing duties: in an old people’s homes and geriatric wards (in the first sub-study in 2011, *n* = 56); in home service, public healthcare units, and community hospital wards (in the second sub-study in 2012; *n* = 80); and on university hospital wards (in the third sub-study in 2013, *n* = 83) in the city of Tampere, Finland. The study protocol and time frame of each identical sub-study are presented in the study protocol [42]. The eligibility criteria, recruitment of participants, and reasons for exclusion have been described in detail previously [41–43]. Briefly, 30–55-year-old female healthcare workers were eligible if they had worked in their current job for at least 12 months and had experienced LBP of an intensity 2 or above on a numeric rating scale (NRS; 0–10) [44] within the preceding four weeks. Age range was set to get a study sample, which participants had been exposed to physically demanding work, and would still be working during the 24 months’ follow up (in NURSE RCT). The exclusion criteria were a serious earlier back injury (disc protrusion, fracture, surgery), chronic LBP as diagnosed by a physician or a self-report of continuous LBP over the past seven months or longer, pregnancy or recent delivery (< 12 months), and engaging in a neuromuscular type of exercise more than once a week.

At the pre-study screening, the mean LBP intensity, measured on a numeric rating scale of 0–10, was 4.7 (SD 1.8) [43]. Most of the study subjects (82%) experienced LBP on some or most days of the week but not daily, and 18% experienced LBP daily [43]. Duration of LBP was less than 3 months for 65% [43]. According to definitions made by Kongsted et al. [4], the majority of the study sample could be described as suffering from sub-acute, mild to moderate, recurrent, or fluctuating non-specific LBP. Although term “recurrent LBP” lacks consensus [45], we use it to describe the study subjects, most of whom had a recurring pain behaviour [43] .

**Fig. 1:**
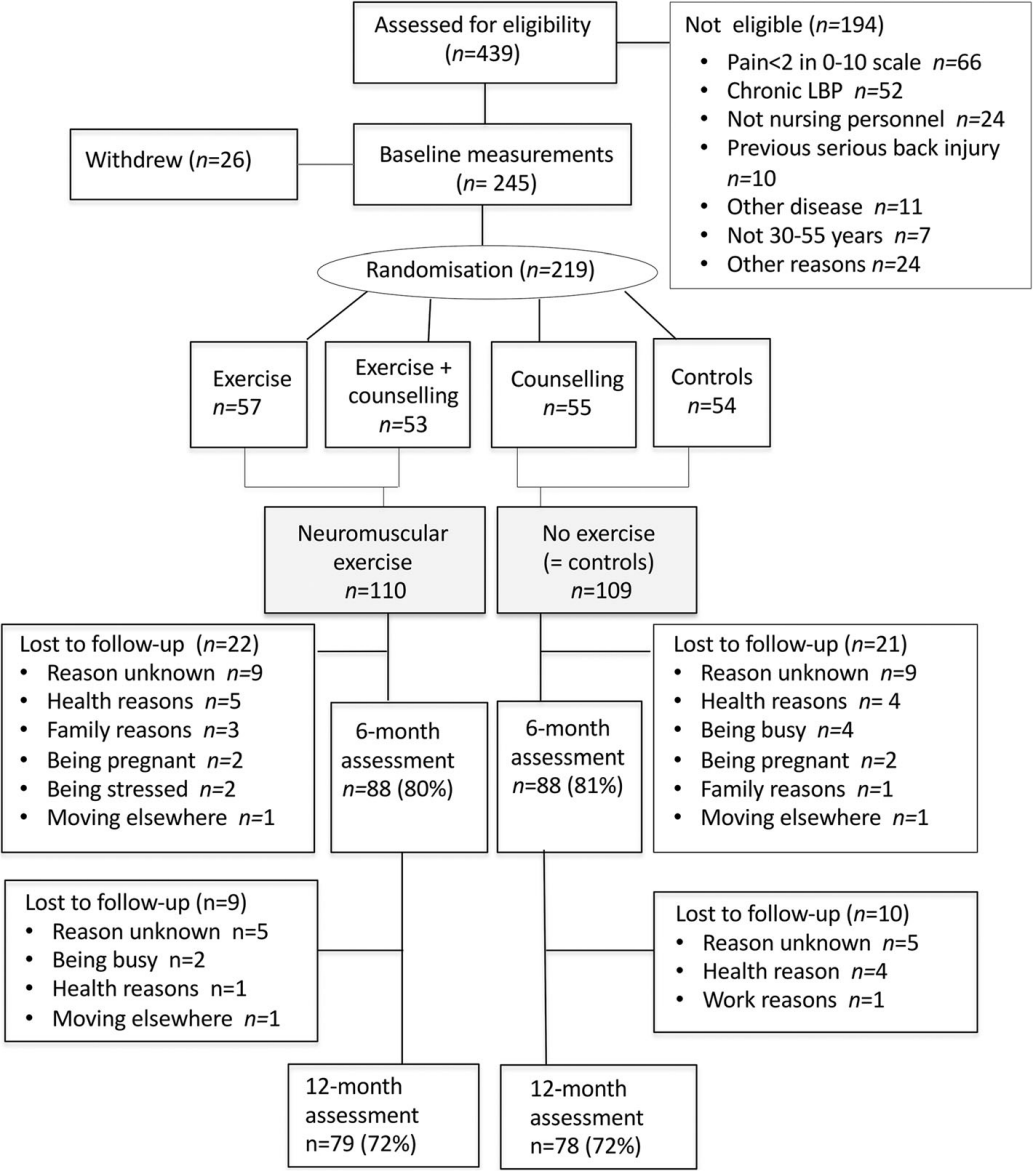
Trial profile (CONSORT flow chart)

The sample size of at least 160 subjects was estimated for the primary outcome of intensity of LBP on Visual Analog Scale 0–100 [42]. The present study is a secondary analysis of the NURSE RCT. The aim is to investigate in detail the effects of the neuromuscular exercise programme on LBP intensity, pain interfering with work, lumbar movement control, physical fitness, and work-related factors in participants randomly assigned to an exercise group or non-exercise control group, regardless of receiving back care counselling in the NURSE RCT (50% of each group, exercise or non-exercise, received counselling). The study design and grouping of the participants are shown in Fig. 1.

### Measurements

Measurements were taken at the baseline, immediately after the intervention at 6 months, and at 12 months from the baseline at the UKK Institute for Health Promotion Research in Tampere, Finland. Experienced, specially educated personnel who were blinded to group allocation and not involved in the interventions conducted all the measurements. The outcome measurements are presented in Table 1.

**Table 1:** Outcome measurements of the study

	Measurement
*Primary outcome:*	
Low back pain	Pain intensity: Visual analogue scale (VAS; 0–100 mm) during past month [46] (0 = no pain, 100 = worst possible pain)
*Secondary outcomes:*	
Pain interfering with work	Subscale from the RAND 36 Health Survey [47]; 0–100 (0 = worst pain and extreme difficulties, 100 = no pain and no difficulties)
Movement control of the low back	MCI test battery [48] consisting of four tests: 1) the waiter’s bow (flexion of the hips in the upright standing position without movement of the lower back), 2) dorsal tilting of the pelvis, 3) sitting knee extension, and 4) prone-lying active knee flexion [49]
Physical fitness:	
Aerobic fitness	6MWT; maximal walking distance (metres) in 6 min [50]
Muscular strength and endurance	Modified push-ups [51], dynamic sit-ups [52], one-legged squats [51]
Work-related factors:	
Work-induced lumbar exertion	Perceived exertion in the low back after a typical working day [53]. NRS 1–5; 1 = no exertion … 5 = high exertion. Ratings were split into two groups: 1 + 2 = no exertion, 3–5 = moderate to high exertion
Physical functioning in nursing tasks	Ability to manage with heavy, task-specific nursing duties, including patient transfer: Sum score of NRS 0–10 with eight selection points: 0 = no difficulties … 80 = does not manage at all [42]
Tiredness, sleepiness, and difficulties in recovering from work	Sum score from four questions: 4 = no tiredness or sleepiness and recovering well from work … 18 = long-term, daily tiredness and sleepiness, and not recovering from work [53]

*6MWT* six-minute walk test, *MCI* movement control impairment, *NRS* numeric rating scale, *VAS* visual analogue scale

The repeatability of the physical fitness tests and lumbar MCI tests used with this study sample was confirmed in the first sub-study (*n* = 47) [49]. From the original MCI test battery of 6 tests [48], two tests with poor repeatability (rocking forwards and backwards and 1-leg stance) were removed (Table 1.). A precise description of those tests is given in the repeatability article’s supplement [49].

### Randomisation

A method of sequentially numbered sealed envelopes was used in all three sub-studies of the NURSE RCT to assign the participants to the four study groups. Once a participant had consented to enter the study at the baseline measurement, the next envelope in order was opened and the participant was then offered the allocated study group (exercise + counselling, exercise only, counselling only, and controls) [41, 42]. In the analysis of the present study, the first two mentioned groups (exercise + counselling, and exercise only) were merged to be the “exercisers”. The latter two groups (counselling only and controls) were merged to be the “non-exercisers”, i.e. the control group.

### Exercise intervention

The overall aim of the 6-month exercise programme was to reduce pain-induced disturbances of movement control and increase the muscular strength and endurance needed in heavy nursing tasks [42]. The focus was on controlling the neutral spine posture in gradually progressive exercises. The learning objectives for the first two months were to learn the right performance technique, control the neutral spine posture during low-load exercises, and combine breathing with each exercise [42]. During the second and third stages (months 3–4 and 5–6, respectively), the programme was progressive in terms of the demands for coordination, balance, and muscular strength and endurance [42]. The aims and content of the NME programme are presented in the protocol article’s Additional file 1 [42]. Briefly, the general training principles and objectives were: 1) to increase spinal stability using exercises that minimise the load on spinal structures but induce a high level of muscular activity [54–59]; 2) to improve the endurance of the trunk musculature [58]; 3) to improve balance [60], postural control [61], and light co-contraction of the stabilising muscles around the lumbar spine in various upright postures and movements [62]; 4) to combine breathing with exercises, and thus take advantage of the spine-supporting role of the increased intra-abdominal pressure [63, 64]; 5) to increase the muscular strength of the lower limbs in functional squatting movements [65]; and 6) to achieve a normal range of motion in the spine, especially in the thoracic region and the hip and ankle joints [42]. The exercises are presented in Additional file 1.

The goal was to exercise twice a week in supervised NME classes (lasting 60 min) for the first two months, and in one supervised class and one home session – with help of a DVD (lasting 50 min) or booklet produced for the study – per week for the following four months. Supervised exercise groups were organised near the workplaces of the healthcare workers from Monday to Friday, starting 15 min after the typical work shifts ended [42].

The instructors of the NME groups were all certified Pilates instructors with a background education in physiotherapy, a masters’ degree in health sciences, or both. Education about the standardised exercise programme in three progressive stages was organised for the instructors by AT before the intervention and before moving to the next progressive stage in each consecutive sub-study. The traditional key principles of the Pilates method – i.e., concentration, centering, control, precision, breathing, and flow [66] – were followed, with a special emphasis on intrinsic feedback of the posture of the spine in each exercise in order to discriminate the movement of the lumbar spine from the movement of the hip joints and thoracic spine [67, 68]. To avoid any contamination with back care counselling intervention (in the original 4-arm setting of the NURSE RCT), the instructors were advised to follow the standardised exercise programme, and to avoid other kind of counselling (like physical activity and other lifestyle). Individual modifications to the standardised NME program were sometimes needed because of musculoskeletal problems other than LBP. The participants were asked to report any increase in back pain during or after the exercise sessions.

Two instructed exercise sessions were provided to the participants of the exercise group during the follow-up time (from 7 to 12 months).

### Statistical analysis

Power calculations were conducted based on the original NURSE RCT four-arm study design [42]. The sample size was estimated for the primary outcome of pain intensity (on a visual analogue scale; VAS), with an emphasis on the proportion (%) of patients with improved LBP on the VAS (0–100) [42]. It was expected that there would be a minimal difference of 20% between the intervention groups in the proportion of patients with an improved VAS (at least 15 mm, which indicates the minimal clinically important change) [46]. In order to detect a difference in main effects between groups with a significance level of 0.05 and a power of 80%, at least 160 participants were needed for the study. For the compensation of the probable loss of participants in the follow-up, the aim was to recruit a total of 240 participants [42].

The descriptive results at baseline are presented as means with standard deviations (SD) or proportions. The differences between the two groups at the baseline were analysed by the Independent samples *t*-test, χ^2^ test, or Mann–Whitney *U* test as applicable. The results of the intervention were analysed according to intention-to-treat (ITT) principle. Differences in time (at the three measurement points) between the two groups (exercisers vs non-exercisers) were tested using a generalised linear mixed model (GLMM). All analyses were adjusted to take the effect of counselling into consideration. Other potential confounding factors were background variables (age, hormonal status, BMI, sub-study and civil status), work- and health-related factors (shift work/regular work, perceived health, blood pressure, tiredness and sleepiness, and current medication), fitness components, and self-reported physical activity. Only those confounding factors that improved the model in the second stage in the sense of Bayesian information criteria were included in the final GLMM.

For the per-protocol (PP) analysis, the study sample was assigned to two groups in order to investigate the effectiveness of the exercise. Those who exercised at least once a week were assigned to the exercise group, and the reference group consisted of those who exercised less than once a week and the controls. The same GLMM models were used for the PP and ITT analyses.

The correlation between the change in LBP intensity and the change in the results of other measurements after the intervention period were calculated by Pearson (r_p_) or Spearman’s correlation coefficient (r_s_) as applicable. More accurate analyses of the changes in lumbar movement control according to the baseline results were analysed with the χ^2^ test. All statistical analyses were conducted using IBM SPSS statistics software (IBM SPSS Statistics for Windows, Version 25.0. Armonk, NY: IBM Corp.).

## Results

A total of 219 women underwent randomisation from October 2011 to August 2013. Of the 219 women, 80% (*n* = 176) participated in the 6-month follow-up measurements immediately after the intervention period and 72% (*n* = 157) participated in the 12-month follow-up measurements [41]. The drop-out rate was equal in both study groups (Fig. 1).

The participant characteristics are presented in Table 2. The participants’ mean age was 46 years, and they had worked in their current job on average for 11 years. Of the participants, 87% were nurses or nursing assistants, and 70% did shift work. The descriptive results of the outcome measures are presented in Table 3. At baseline, the BMI was higher (*p* = 0.05) and the results of the modified sit-up tests were lower (*p* = 0.02) in the exercise group (Tables 2 and 3). There were no other group differences.

**Table 2:** Background characteristics of the participants by the study group

	Pilates-type NME group (*n* = 110)		Controls (no-exercise) (*n* = 109)		Missing	*p*-value
	%	Mean (SD)	%	Mean (SD)		
Age, years		46.2 (6.8]		46.6 (6.8)		0.69
BMI		27.0 [4.7)		25.8 [4.0]	2	0.05
Smoking						0.60
daily	15.5		17.4			
occasionally	10.0		13.8			
non-smoker	74.5		68.8			
Civil status: married/cohabiting	60.9		68.8			0.22
Education: secondary school or less	27.5		26.9		2	0.50
Occupation						0.31
nurse	51.0		42.0			
nursing assistant	39.0		42.0			
other (PT, midwife, radiographer)	10.0		16.0			
Number of working years		11.9 [9.2)		10.7 [8.1)	2	0.25
Working times					1	0.66
Regular work	32.0		29.0			
shift work	68.0		71.0			
Perceived health					1	0.31
average or below average	41.0		34.0			
better or much better than average	59.0		66.0			
Perceived fitness in comparison to persons of the same age and gender					1	0.71
lower or much lower	29.0		27.0			
similar	47.0		53.0			
higher or much higher	24.0		20.0			
Number of musculoskeletal pain sites		3.3 (1.2)		3.1 (1.4)		0.31
Depression; PHQ-9 (0–27)		7.9 [4.9)		7.0 (4.3)	1	0.14
High blood pressure: yes	15.5		12.0			0.46
Current use of medication: yes	52.7		57.9			0.44

*BMI* body mass index, *NME* neuromuscular exercise, *PHQ-9* modified Finnish version of the Patient Health Questionnaire, 9 items measuring depressive symptoms [69]

**Table 3:** Baseline characteristics of pain, movement control of the low back, physical fitness, and work-related factors

	Pilates-type NME group (n = 110)		Controls (no exercise) (n = 109)		Missing	*p-*value
	%	Mean (SD)	%	Mean (SD)		
VAS: intensity of LBP (0–100)		36.3 (22.0)		36.0 (23.4)	1	0.94
Bodily pain interfering with work (0–100)^a^		61.5 (18.7)		64.4 (19.3)	8	0.28
MCI sum (0–4)		1.0 (1.0)		1.1 (1.0)		0.94
Deficiencies in MCI test battery						0.91
0	35.5		33.0			
1	35.5		35.8			
2–4	29.1		31.2			
Fitness components:						
6MWT		619.4 (50.4)		624.0 (48.3)	1	
Modified sit-ups		17.2 (4.6)		18.4 (3.7)	1	0.02
% reaching the maximum of 20	61.5		75.2			0.03
One-legged squats		9.4 (2.7)		9.5 (2.4)	3	0.72
Modified push-ups		9.0 (3.4)		9.0 (2.8)	6	0.94
Work-related factors:						
Difficulties in patient handling (0–80)		6.0 (4.9)		6.6 (5.2)	13	0.43
Work-induced lumbar exertion						0.45
little exertion	26.9		31.5		3	
moderate to high exertion	73.1		68.5			
Tiredness and recovery from work (4–18)		10.4 (3.4)		10.0 (3.2)	1	0.20

*6MWT* six-minute walk test, *MCI sum* sum score of movement control impairment tests, *NME* neuromuscular exercise. ^a^0 = worst possible pain and extreme difficulties, 100 = no pain and no difficulties

### Compliance with exercise

The target was to exercise twice a week for 24 weeks, i.e. to complete 48 sessions. The instructors monitored participation in the supervised group exercise, and study subjects kept an exercise diary for their home practice. The mean attendance rate was 26.3 (12.2) exercise sessions, and 53% of the participants exercised 1–2 times a week.

### Effectiveness of the neuromuscular exercise programme

**Fig. 2:**
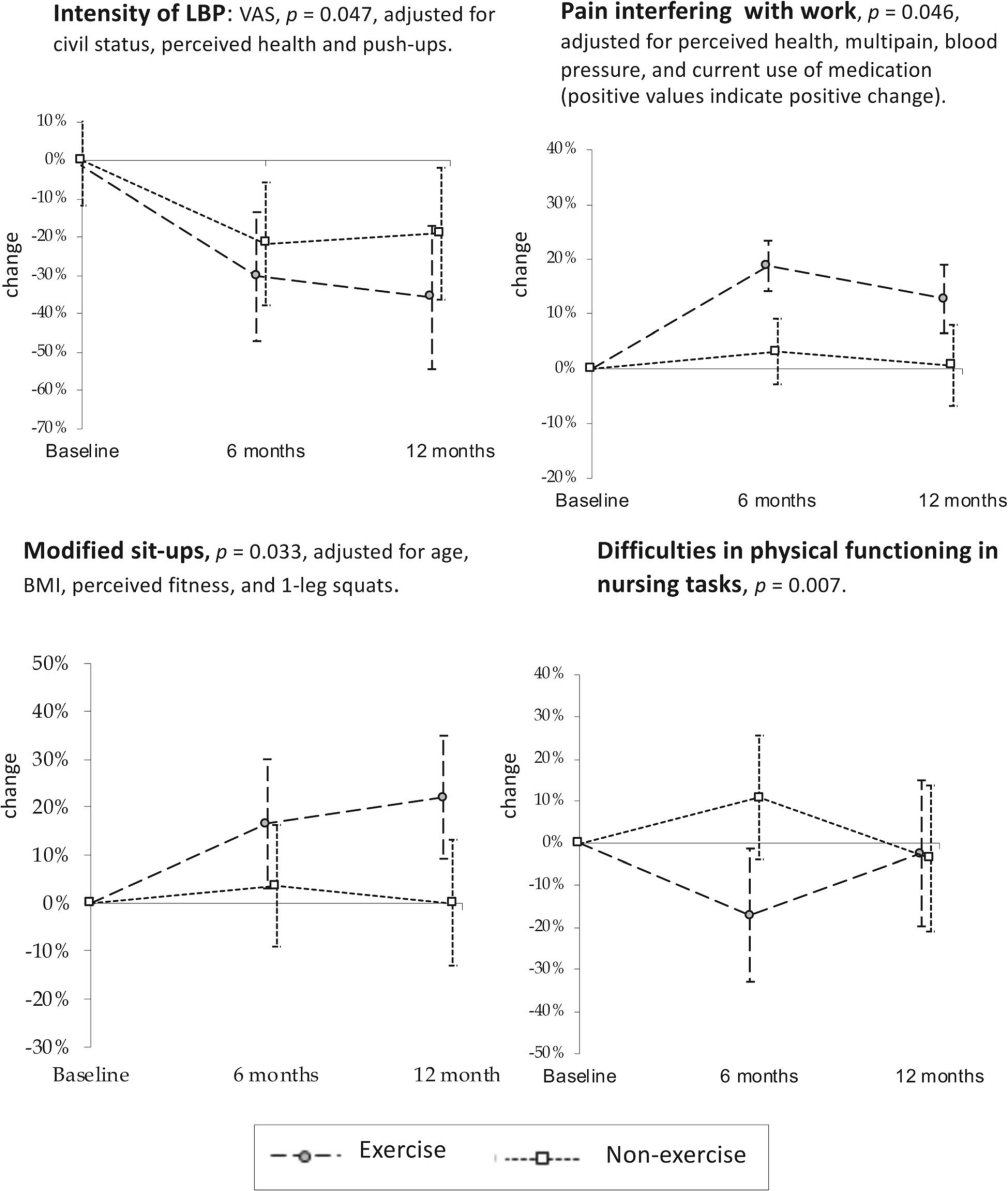
Mean percentage change (95% confidence interval) at 6 and 12 months from baseline

**Fig. 3:**
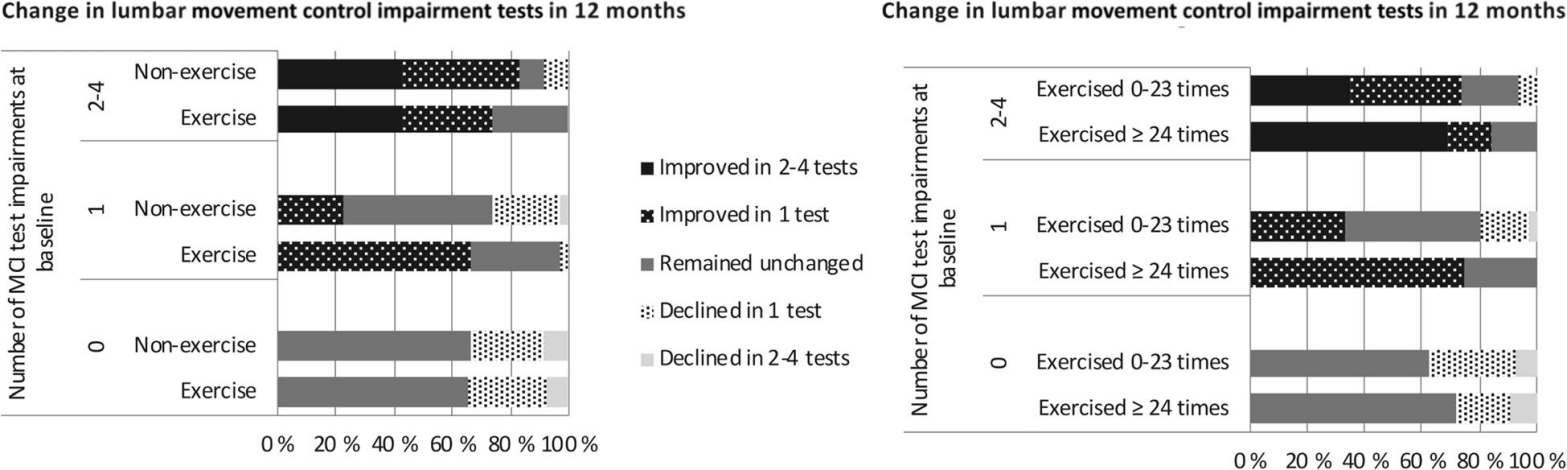
Change in lumbar movement control (MCI) test results from baseline to 12 months. **a** Changes among exercisers (*n* = 79) and non-exercisers (*n* = 78), and (**b**) among exercise compliers (exercised ≥24 times, *n* = 52) and a combined group of less exercised and non-exercisers (exercised 0–23 times, *n* = 105)

The results of the exercise intervention according to the ITT analysis are presented as the mean difference with SD, or as percentages at 6 and 12 months in relation to the baseline (in Table 4). The main results are depicted graphically as the percentage change with 95% confidence intervals at 6 and 12 months in Fig. 2. Changes in lumbar movement control from the baseline to 12 months are described graphically in Fig. 3. The results of the effectiveness of the exercise programme according to the PP analysis are presented in Table 5; we decided not to show statistically non-significant results.

**Table 4:** Difference between the groups at the 6- and 12-month follow-ups in relation to baseline, adjusted for age, perceived health, multisite pain, blood pressure, current use of medication, fitness, and civil status

						Difference in relation to baseline											
	Baseline					6 months					12 months						
	Pilates-type NME *n* = 110		Controls *n* = 109		M	Pilates-type NME *n* = 88		Controls *n* = 88		M	Pilates-type NME *n* = 79		Controls *n* = 78		M	*p*-value	*p*-value, adjusted
	%	Mean (SD)	%	Mean (SD)		%	Mean (SD)	%	Mean (SD)		%	Mean (SD)	%	Mean (SD)			
VAS (0–100)		36.3 (21.9)		36.1 (24.0)	1		−10.7 (24.0)		−6.6 (26.1)	14		−11.3 (21.8)		−6.1 (28.1)	8	0.076	0.047
Pain interfering with work (0–100)		61.5 (18.7)		64.4 (19.3)	8		11.0 (18.0)		2.6 (19.3)	14		7.2 (18.1)		1.3 (25.5)	9	0.035	0.046
MCI (0–4)		1.1 (1.0)		1.1 (1.0)	–		−0.5 (1.0)		−0.3 (1.0)	–		−0.5 (1.0)		−0.2 (1.1)	–	0.036	0.042
*Fitness components:*																	
6MWT (metres)		614 (50.8)		620 (48.8)	1		21.5 (35.4)		8.9 (34.4)	2		22.7 (32.5)		13.7 (36.4)	6	0.075	0.273
Modif. push-ups		9.1 (3.4)		9.0 (2.8)	6		1.7 (2.0)		1.6 (1.9)	10		2.4 (2.6)		2.5 (2.2)	13	0.862	0.979
Sit-ups; % reaching 20 repetitions max.	62		75		1	74		76		1	77		77		2	0.016	0.033
One leg squats (0–12)		9.4 (2.7)		9.5 (2.4)	4		0.4 (1.5)		0.6 (1.4)	10		0.3 (1.7)		0.5 (1.3)	20	0.631	0.420
*Work-related factors:*																	
Work-induced lumbar exertion					3					16					10	0.086	0.138
little	27		32			51		44			48		36				
moderate to high	73		68			49		56			52		64				
Physical functioning in nursing tasks (0–80)		6.1 (4.9)		6.6 (5.2)	11		−0.9 (4.4)		0.3 (4.9)	26		−0.3 (4.8)		−0.6 (4.7)	27	0.007	0.061
Tiredness and recovery (4–18)		10.4 (3.4)		10.0 (3.2)	1		− 0.7 (2.5)		− 0.3 (2.3)	13		−0.3 (3.2)		0.4 (2.3)	7	0.061	0.180

*6MWT* 6 min. Walk test, *M* missing, *MCI* movement control impairment, *NME* neuromuscular exercise, *VAS* visual analogue scale

**Table 5:** Efficacy of the Pilates-type neuromuscular exercise programme: difference in relation to baseline between once a week or more exercised (≥24 exercise sessions) and a combined group of less exercised and controls (≤23 exercise sessions + controls), adjusted for perceived health, BMI, fitness, education, and civil status

			Difference in relation to baseline					
	Baseline		6 months		12 months			
	More exercised *n* = 58	Less exercised + controls, *n* = 161	More exercised, *n* = 58	Less exercised + controls, *n* = 118	More exercised, *n* = 52	Less exercised + controls, n = 105		
	Mean (SD)	Mean (SD)	Mean (SD)	Mean (SD)	Mean (SD)	Mean (SD)	*p*-value	*p*-value, adjusted
VAS (0–100)	36.8 (20.0)	35.9 (23.6)	−15.4 (21.7)	−5.0 (26.1)	−12.7 (22.6)	−6.67 (26.1)	0.057	0.029
MCI (0–4)	1.0 (1.1)	1.1 (1.0)	−.05 (1.0)	−0.3 (0.9)	−0.5 (1.0)	−0.2 (1.0)	0.016	0.017
6MWT (metres)	623 (43.8)	615 (51.8)	27.3 (32.9)	9.3 (35.2)	25.9 (36.5)	14.5 (33.3)	0.020	0.065

*6MWT* 6 min. Walk test, *BMI* body mass index, *MCI* movement control impairment, *VAS* visual analogue scale

### Pain intensity and pain interfering work

At the baseline, the mean pain intensity measured by VAS (0–100) was 36.2 (SD 22.6) [43]. The mean reduction in the exercise group was − 10.7 mm (24.0) at 6 months and − 11.3 mm (21.8) at 12 months compared to − 6.6 mm (26.1) and − 6.1 mm (28.1), respectively, in the non-exercise group (Table 4). The percentage reduction in pain in the exercise group was 30.3% at 6 months and 35.7% at 12 months. The corresponding reductions in the non-exercise group were 21.8 and 19.1%, respectively (*p =* 0.047; Fig. 2).

In the PP analysis, the difference in pain reduction was greater in the more exercised group (*p* = 0.029); the mean reduction at 6 months among the more exercised was − 15.4 mm (21.1), i.e. a reduction of 43.0%. This compares to a reduction of − 5.0 mm (26.1) in the less exercised and non-exercisers, i.e. a reduction of 13.7% (Table 5).

When compared to the results of the non-exercise group, pain interfering with work decreased in the exercise group (*p* = 0.035; Table 4 and Fig. 2). Exercising more did not improve the result in the PP analysis. The participants did not report any adverse events, i.e. an increase in back pain during or after the exercise sessions.

### Lumbar movement control impairments

After the intervention, lumbar MCI decreased more in the exercise group compared to the non-exercise group (*p* = 0.046; adjusted for education level and one-legged squats; Table 4). At the baseline, 35% of the exercise group had no deficiencies in any of the four movement control impairment tests, 35% had impairments in one test, and 29% had impairments in 2–4 tests (Table 3). The corresponding percentages in the non-exercise group were 33, 36, and 31%, respectively. In the exercise group, of those who had any impairment at the baseline, 68% improved their result, 30% remained unchanged, and 2% were more impaired at 12 months (Fig. 3). The corresponding percentages in the non-exercise group were 46, 39, and 15%, respectively. In the PP analysis, the decrease in MCI was more obvious in the more exercised compared to the less exercised and the non-exercisers (*p* = 0.017; Table 5 and Fig. 3).

The increase in lumbar movement control did not correlate with the decrease in pain intensity at either 6 months (r_s_ = 0.03, *p* = 0.75) or 12 months (r_s_ = 0.07, *p* = 0.42).

### Fitness components

Compared to the non-exercisers, abdominal strength increased in the exercisers (*p* = 0.02). No significant differences between the study groups were found regarding any other fitness components in the ITT analysis (Table 4). The increase in abdominal strength did not correlate with a decrease of pain at either 6 months (r_s_ = − 0.10, *p =* 0.09) or 12 months (r_s_ = − 0.15, *p =* 0.07).

In the PP analysis, the more exercised increased their walking distance in the six-minute walk test (6MWT) compared to the less exercised and the non-exercisers (*p* = 0.02; Table 5). The reduction in pain intensity correlated with the increase in walking distance at 6 months (r_p_ = − 0.17, *p* = 0.03), but not at 12 months (r_p_ = − 0.06, *p* = 0.46).

### Work-related factors

In the longitudinal analysis, the exercise group perceived fewer difficulties in physical functioning at work (*p* = 0.007) compared to the non-exercise group (Table 4 and Fig. 2). The change was most obvious at 6 months, when the difficulties decreased in the exercise group by 17.1%, while the difficulties increased in the controls by 10.9%. At 12 months, there were no longer group differences (Fig. 2). After adjustments (for age, multisite pain, self-reported physical activity, modified push-ups, and tiredness and sleepiness), the result was not statistically significant. The decrease in difficulties correlated with a decrease in pain intensity at 6 months (r_s_ = 0.27, *p* = 0.001). The exercise group seemed to perceive less tiredness and better recovery from work (*p* = 0.06), and less work-induced lumbar exertion (*p* = 0.09) compared to the non-exercisers (Table 4), but the differences were not statistically significant in either the ITT or PP analyses.

## Discussion

The novel finding of the present study was that the modified 6-month Pilates-type NME with focus on controlling the neutral spine posture in gradually progressive stages was effective in reducing LBP intensity, pain interfering with work, and impairments in lumbar movement control among female health care workers with sub-acute or recurrent NSLBP measured at 6 and 12 months from the baseline. The NME intervention also decreased difficulties in physical nursing duties, but it was ineffective in improving fitness components other than abdominal strength compared to the results for non-exercisers. However, the more exercised did gain better results in the reduction of pain intensity, lumbar movement control, and 6MWT.

Although nursing is among the top risk professions for LBP, and although exercise is commonly recommended as treatment for people with LBP, only a few high-quality intervention studies considering exercise for healthcare workers with LBP have been published. In a recent systematic review [70] investigating intervention studies among nursing personnel with LBP, only three RCTs including exercise in the interventions and having a low risk of bias were found. Stretching [71] or combined strength training and stretching [72] decreased pain among nurses with chronic pain, but a programme including counselling, segmental stabilisation, and general exercise was not superior to general exercise alone in reducing pain among nurses with sub-acute LBP [73]. At present there is no strong evidence for the efficacy of any intervention in the prevention or treatment of LBP in nursing personnel [70].

The contents and length of our NME programme focusing on control of the lumbar spine posture differed from the above-mentioned exercise programmes. On the other hand, our results are in line with previous studies emphasising control of a lumbar neutral spine posture in both exercise and counselling conducted among people with strenuous work [25, 62].

Among general population with LBP, lumbar movement control exercises appear to be more effective in reducing pain in short term, and in improving disability in long term. However, the quality of evidence varies from very low to moderate. Based on the available studies, it is difficult to assess the relative effectiveness of lumbar movement control exercises compared to other interventions offered to people with LBP [27].

Exercise is the most effective treatment for the management and prevention of spinal pain [17]. However, knowledge regarding how and why exercise programmes work is somewhat limited. Physical activity and exercise have been shown to activate endogenous pain inhibitory mechanisms and lead to a reduction in sensitivity to noxious stimuli (termed “exercise-induced hypoalgesia”) regardless of the type of physical activity [74–76]. Protective effect of practising regular exercise on developing LBP has recently revealed among healthcare workers [11].

Two common assumptions about LBP are 1) that motions, postures, and loads are responsible for tissue damage or irritation that leads to pain [77] and 2) “risky” movements both during work and also during physical training can eventually result in cumulative tissue damage [78, 79].

Many people with LBP have altered lumbar proprioception [61, 80], and they are probably less “movement aware”, with reduced postural control [80] and altered spinal movement patterns [81]. Placing an emphasis on how participants move (i.e. posture and movement control, performance technique, and alignment) may be an effective training strategy to transfer desirable movement patterns to occupational tasks [78, 82]. Frost et al. [78] compared firefighters assigned to a 12-week programme of movement-guided fitness training, conventional fitness training, or a control group. Both fitness-training groups showed significant improvements in all fitness categories, but only the movement-guided group showed spine and knee motion control when performing different occupational tasks [78]. In addition, a single motor skill training session emphasising intrinsic feedback to decrease early-phase lumbar excursion can result in better lumbar movement control in functional tasks among people with LBP [83]. These results support the argument that exercise can be used to change motor behaviour, provided that movement-oriented feedback is offered when exercising. In a physically demanding job like firefighting or nursing, being physically fit may play a role in the prevention of future injuries, but it is likely insufficient for this purpose on its own [78]. The way in which movements are controlled and coordinated influences musculoskeletal loading [78].

The exercise programme in the present study included exercises targeted at increasing the strength and endurance of the torso muscles, but we detected significant changes only in abdominal muscle strength. In the PP analysis, the more exercised improved their walking distance in the 6MWT compared to the less exercised and the controls. The exercise programme was not targeted at improving aerobic fitness. Thus, the result can be explained by either the reduction of pain or increased hip and/or thoracic spine mobility (which were practiced in the exercise group, but not measured in the study).

In the exercise programme, special emphasis was placed on movement control, posture, and breathing, which are considered important when applying Pilates exercises for people with LBP [84]. A focus on breathing is one special feature that distinguishes Pilates-type exercise from conventional exercise programmes. There is low to moderate evidence that breathing exercises can reduce pain in chronic NSLBP [85]. In the practice of Pilates, the breathing technique is called lateral breathing, and the exercises are conducted at the pace of each participants’ calm breathing tempo [66]. This technique was also followed in the present study. The possible effects of this kind of technique on pain remain unclear due to the lack of measurements.

In the literature, standardised exercise programmes for people with LBP are criticised for presenting the idea that a “one size fits all” approach is appropriate for a multifactorial problem like LBP [17]. The current opinion emphasises the bio-psychosocial nature of LBP, where comorbidities and lifestyle factors also play an important role [86]. In the original NURSE RCT with the four-arm setting, the back-care counselling intervention was more concerned with psycho-social and lifestyle factors [41, 42]. The combined exercise and back-care counselling intervention was also more effective in reducing LBP intensity and sickness absence than exercise alone [41].

In general, the NME programme used in the present study was feasible and the biomechanical principles can be modified into other kind of exercise training. This NME program can be recommended specially for those who are interested in Pilates- or yoga-type NME, but the exercises can be tailored according to patient’s preferences to improve exercise adherence. The NME program improved several measurement variables and reduced pain compared to no exercise in the early rehabilitation of a sample who had non-chronic low back troubles and were at risk for chronic pain due to physically burdensome work [12, 87]. Many European countries are facing shortages of healthcare workers, and decreased work ability is an important determinant of leaving the nursing profession [88]. Therefore, interventions targeted at risk factors causing LBP and the early rehabilitation of LBP among healthcare workers are needed. In this study, we presented one type of effective, feasible exercise programme, but we cannot say that it is superior to any other exercise type.

### Limitations of the study

The main limitations of the study relate to the measurement methods and only moderate exercise compliance.

Lumbar movement control was assessed by a battery of four, repeatable MCI tests [49], (waiter’s bow, pelvic tilt, sitting knee extension, prone knee flexion), but the test battery is probably not sensitive enough to detect all (or the smaller) changes in movement control. The four tests measure lumbar movement control principally in the sagittal plane, not in the frontal or horizontal plane, which are essential in both walking and performing nursing duties that often involve standing in asymmetric poses.

We used field tests to measure physical fitness. Smaller changes in muscular strength and endurance cannot be detected with the tests used. With the measurement methods used in the study, we cannot define which elements of the exercise programme caused the reduction in pain. The reason for the pain reduction could be regular exercise in itself, learning to control the movement of the lumbar spine, strengthening the musculature in the torso, or focusing on breathing with the movements – or a combination of all these factors.

Compliance with the exercise regimen was only moderate, which is usual in exercise intervention studies for people with musculoskeletal pain [89]. A training programme of 6 months is quite long in comparison to the duration of 6–12 weeks used in several other studies [39]. Needless to say, only those exercise programmes that are performed can be effective. Thus, a more accurate analysis of the compliance rate and the possible association with baseline factors will be investigated with this study sample in the future. On the other hand, positive changes in several measurement variables were detected with a dose lower than targeted. The compliance rate was probably too low to affect fitness or work- related measurements. A supervised exercise programme of 6 months is also expensive [41], and we do not know if a shorter programme would have been as effective.

## Conclusion

The 6-month modified Pilates-type NME intervention was effective in reducing pain, lumbar movement control impairments, and pain interfering with work; it also improved abdominal strength and physical functioning in nursing tasks among healthcare workers with sub-acute or recurrent LBP compared to not exercising. There was a dose-response for effects on pain intensity and lumbar movement control. The exercise programme was feasible, and its principles can be applied to other kinds of exercise programmes.

## Additional file

Additional file 1: Modified Pilates-based neuromuscular exercise program with focus on controlling the neutral lumbar spine posture (PDF 1593 kb)

## Data Availability

The datasets used and analysed during the current study are available from the principal researcher (JS; jaana.suni@ukkinstituutti.fi) of the NURSE RCT upon reasonable request.

## Abbreviations

6MWT: Six-minute walk test
BMI: Body mass index
GLMM: Generalised linear mixed model
ITT: Intention-to treat
LBP: Low back pain
MCI: Movement control impairment
NME: Neuromuscular exercise
NRS: Numeric rating scale
NSLBP: Non-specific low back pain
PP: Per protocol
RCT: Randomised controlled study
r_p_: Pearson correlation coefficient
r_s_: Spearman’s correlation coefficient
SD: Standard deviation
VAS: Visual analogue scale
