# Supplementary material for: Neuromuscular exercise reduces low back pain intensity and improves physical functioning in nursing duties among female healthcare workers; secondary analysis of a randomised controlled trial
Source: BMC Musculoskelet Disord. 2019 Jul 13;20:328. doi: 10.1186/s12891-019-2678-x (PMC6626624; doi:10.1186/s12891-019-2678-x)

Additional file 1.

Modified Pilates-based neuromuscular exercise program with focus on controlling the neutral lumbar spine posture

Overall target: to reduce pain-induced disturbances of movement control and increase the muscular strength and endurance needed in heavy nursing tasks

In each exercise the participants were instructed to concentrate on (1) the maintenance of neutral spine posture using light co-contraction of the trunk muscles, and (2) neutral alignment between body parts

In all exercises, the target was to combine breathing with exercises, and thus take advantage of the spine-supporting role of the increased intra-abdominal pressure.

In warm-up, light so called pre-Pilates exercises were performed. Also flexibility exercises, presented under the Target number 5, were included.

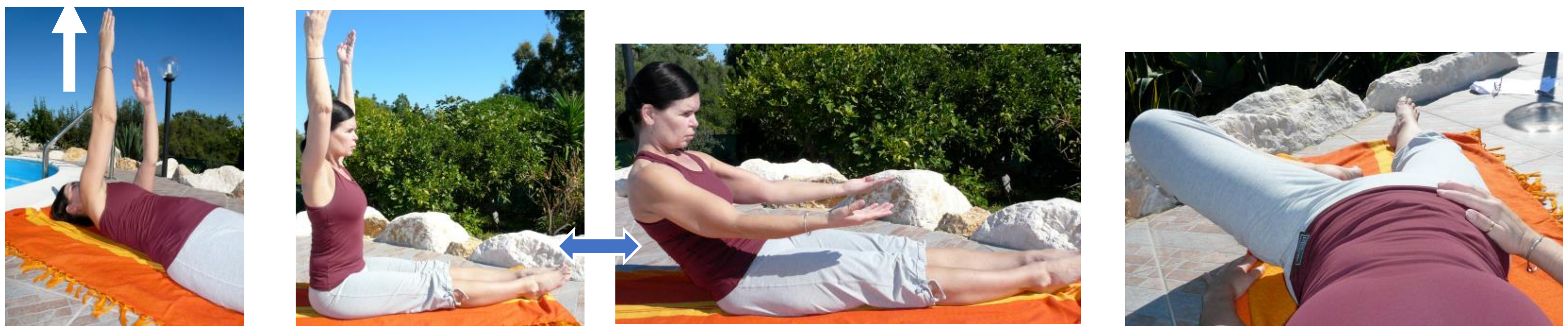

Repetitions and progression:

6-8-10 repetitions. During progression, increasing the range of motion (deeper squats; flexibility exercises) or increasing the holding time were instructed as applicable.

<u>Specific targets and description of the corresponding exercises :</u>

1. To increase spinal stability using exercises that minimise the load on spinal structures but induce a high level of muscular activity

	Stage I	Stage II	Stage III
Modified curl-up ("McGill curl up)	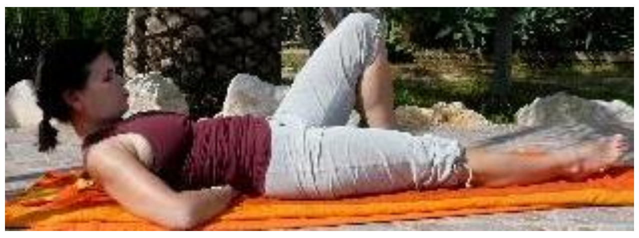	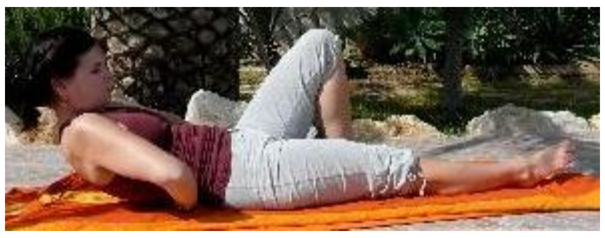	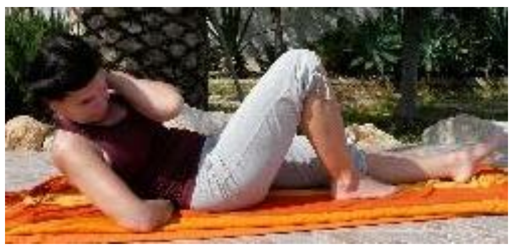
Bird dog	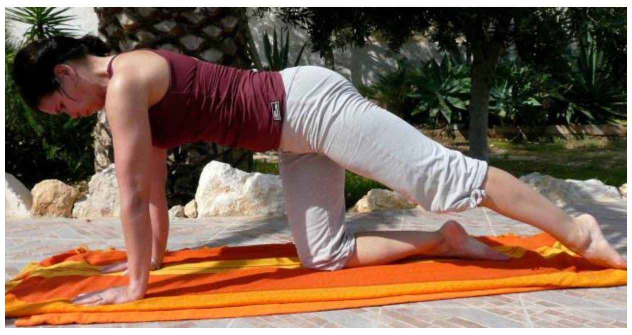	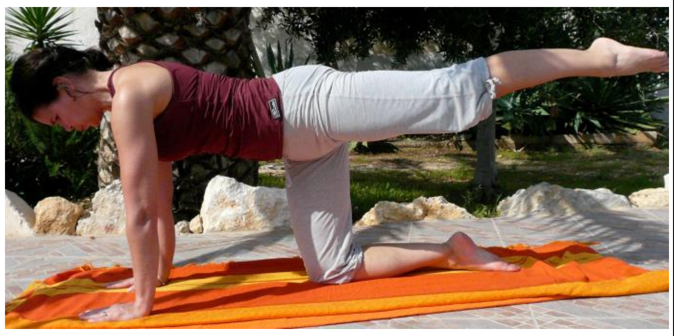	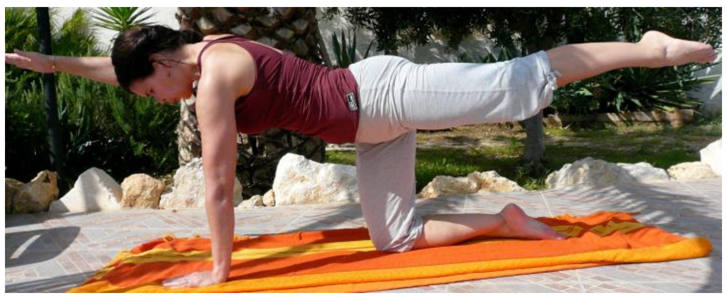
Side bridge or Mermaid	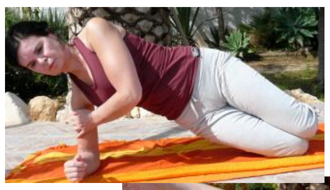 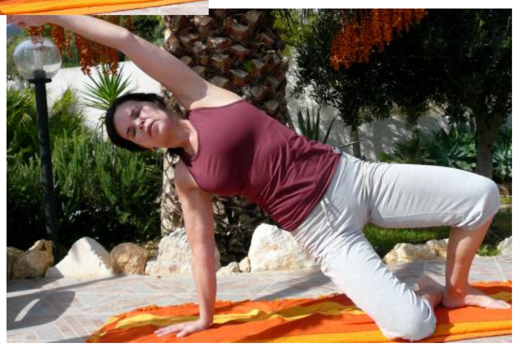	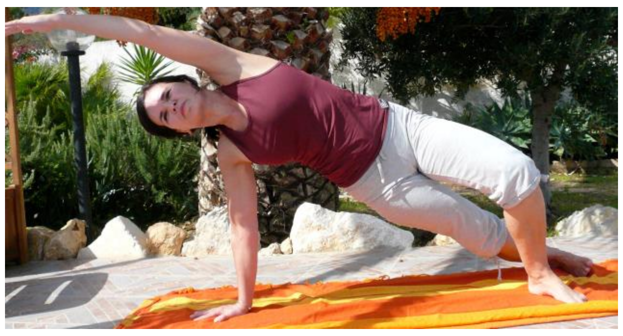	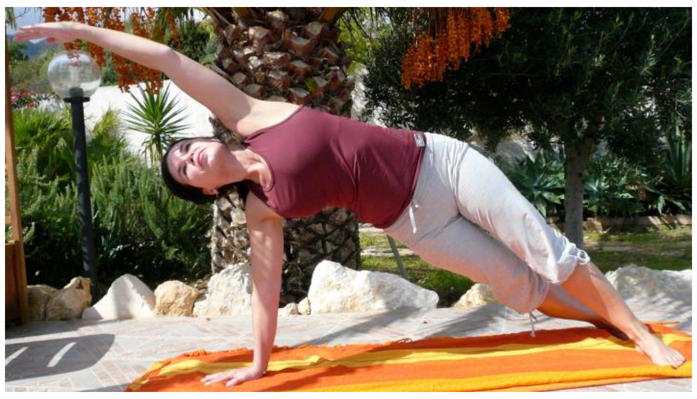

2. To improve the endurance of the trunk musculature

	Stage I	Stage II	Stage III
The 100	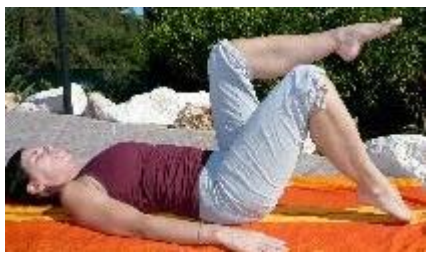	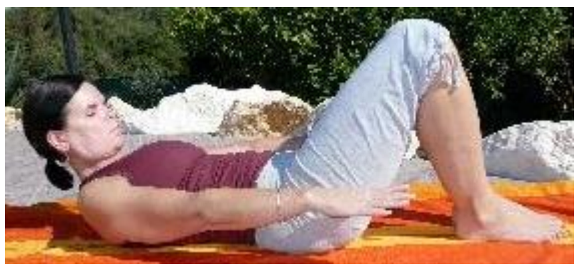	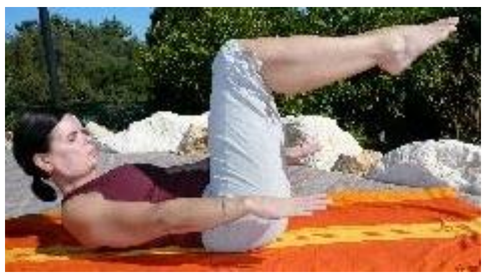
1 leg stretches	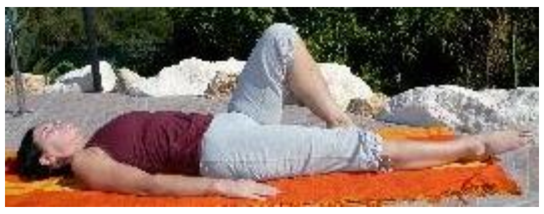	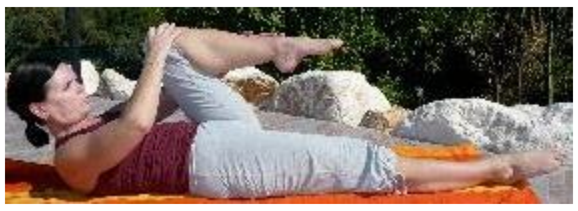	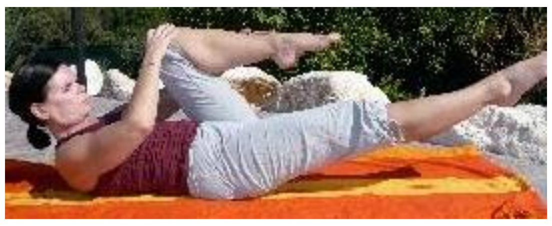
Shoulder bridge with 1 leg lifts	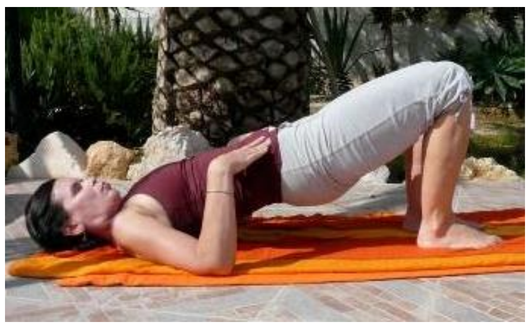	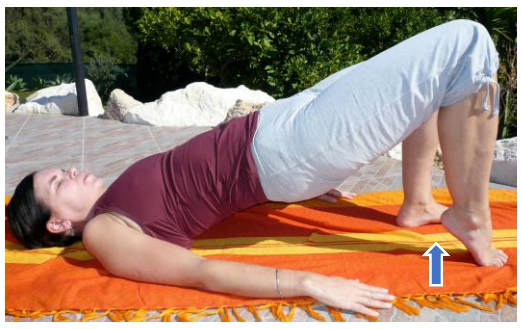	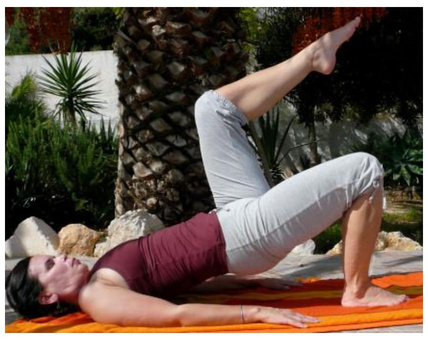
1- and 2-leg lifts	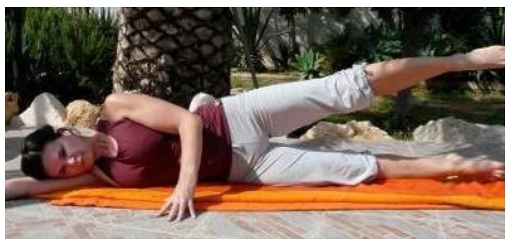	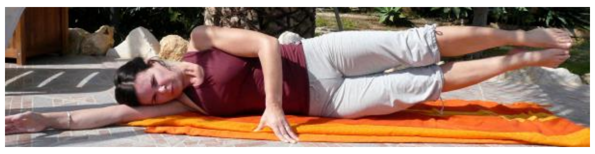	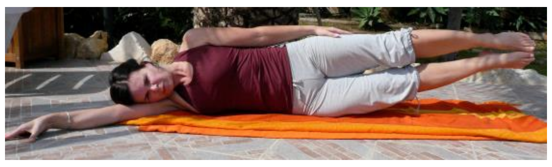
Kobra → 1-leg kicks	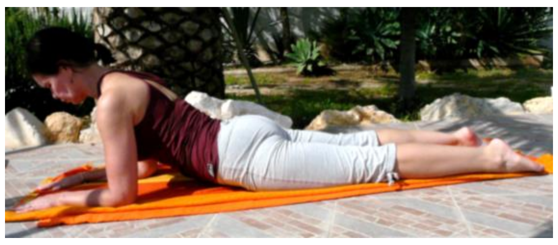	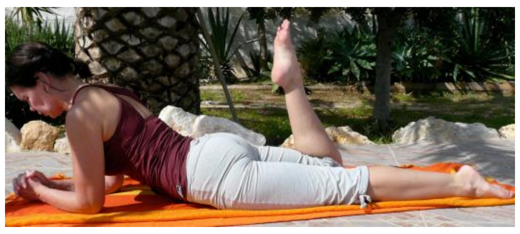	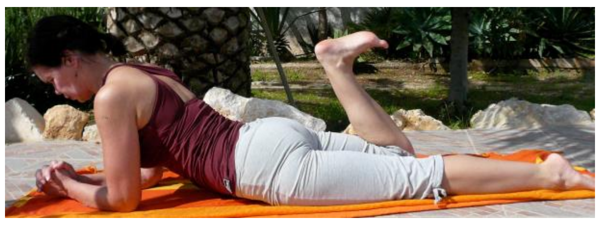
Lifting the knees in 4-point kneeling ; weight shifts forwards and backwaords in plank pose	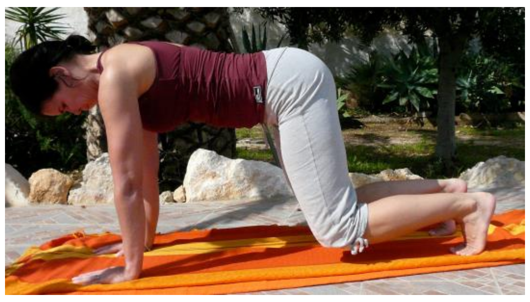	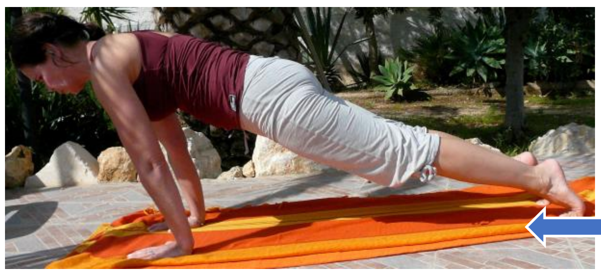	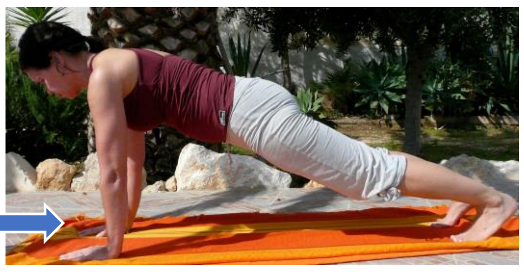
1-leg circles to both directions	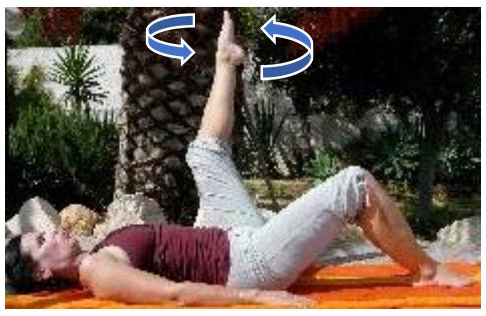	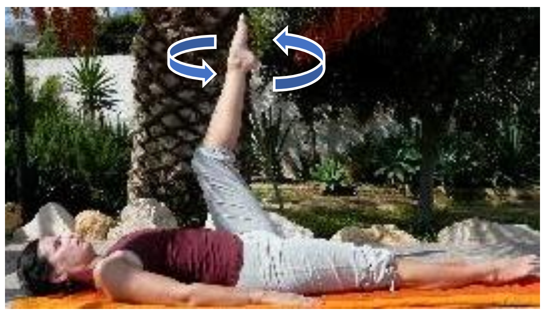	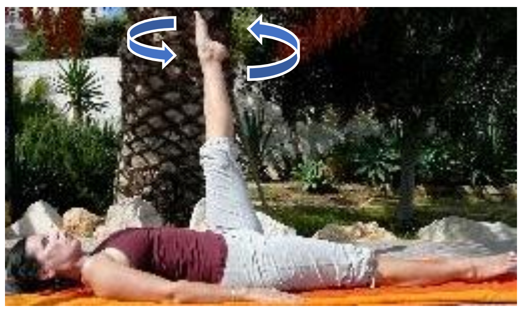

3. To improve balance, postural control and light co-contraction of the stabilising muscles around the lumbar spine

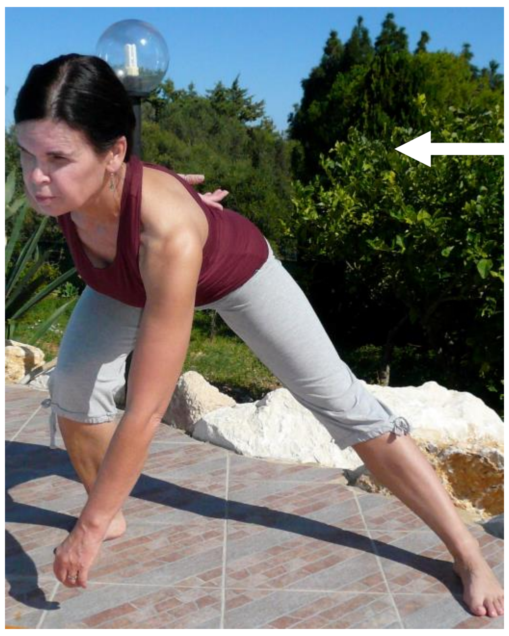
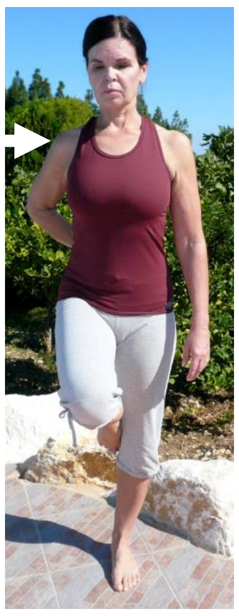
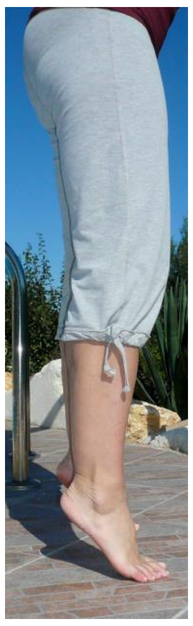

4. To increase the muscular strength of the lower limbs in functional squatting movements

	Stage I	Stage II	Stage III
”Tai chi –warrior”. Progression: coordinative demands with arm movements, deeper squat			

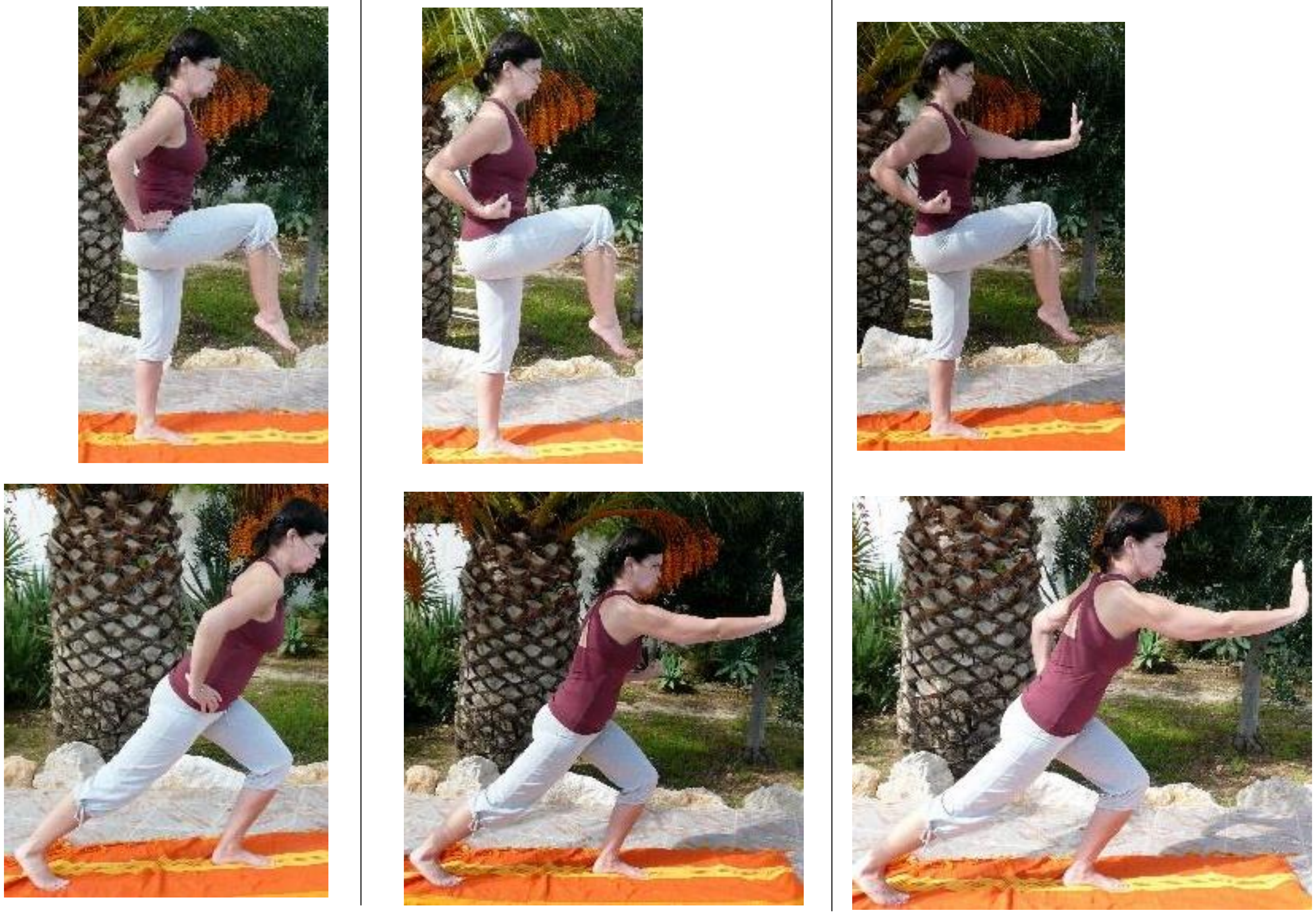

Different squats with weight shifts, arm movements or rotation of upper body

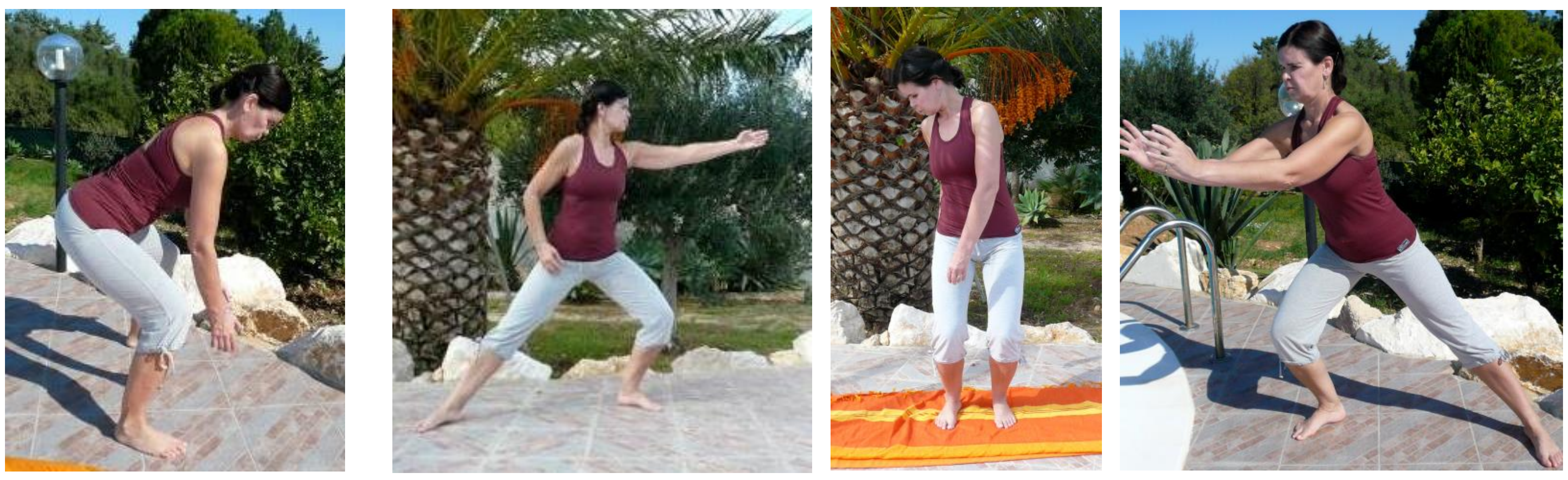

5. To achieve a normal range of motion in the spine, especially in the thoracic region and the hip and ankle joints

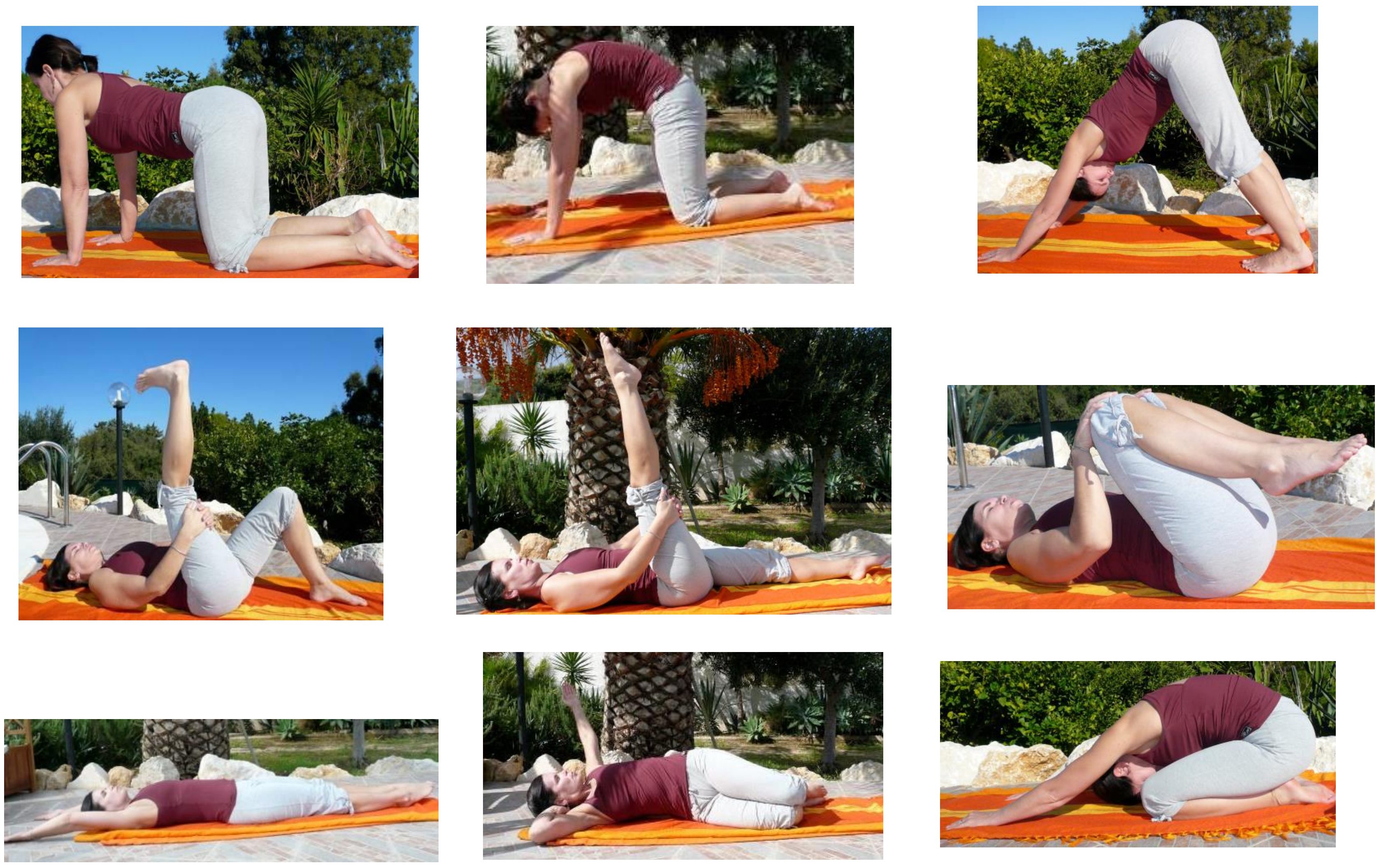

Supplement: Supplementary file 1 — Modified Pilates-based neuromuscular exercise program with focus on controlling the neutral lumbar spine posture (PDF 1593 kb) [file 12891_2019_2678_MOESM1_ESM.pdf]
